# Inhibition of Apoptosis in a Model of Ischemic Stroke Leads to Enhanced Cell Survival, Endogenous Neural Precursor Cell Activation and Improved Functional Outcomes

**DOI:** 10.3390/ijms25031786

**Published:** 2024-02-01

**Authors:** Rehnuma Islam, Jan-Eric Ahlfors, Ricky Siu, Humna Noman, Roya Akbary, Cindi M. Morshead

**Affiliations:** 1 Institute of Medical Science, University of Toronto, 1 King’s College Circle, Toronto, ON M5S 3E1, Canada; 2 New World Laboratories, 275 Boul. Armand-Frappier, Laval, QC H7V 4A7, Canada; 3 Department of Surgery, University of Toronto, 149 College Street, Toronto, ON M5T 1P5, Canada; 4 Institute of Biomedical Engineering, University of Toronto, 164 College Street, Toronto, ON M5S 3G9, Canada; 5 Donnelly Centre for Cellular and Biomolecular Research, University of Toronto, 160 College Street, Toronto, ON M5S 3E1, Canada

**Keywords:** stroke, neural precursor cells, neural stem cells, neuronal survival, neurogenesis

## Abstract

Stroke results in neuronal cell death, which causes long-term disabilities in adults. Treatment options are limited and rely on a narrow window of opportunity. Apoptosis inhibitors demonstrate efficacy in improving neuronal cell survival in animal models of stroke. However, many inhibitors non-specifically target apoptosis pathways and high doses are needed for treatment. We explored the use of a novel caspase-3/7 inhibitor, New World Laboratories (NWL) 283, with a lower IC50 than current caspase-3/7 inhibitors. We performed in vitro and in vivo assays to determine the efficacy of NWL283 in modulating cell death in a preclinical model of stroke. In vitro and in vivo assays show that NWL283 enhances cell survival of neural precursor cells. Delivery of NWL283 following stroke enhances endogenous NPC migration and leads to increased neurogenesis in the stroke-injured cortex. Furthermore, acute NWL283 administration is neuroprotective at the stroke injury site, decreasing neuronal cell death and reducing microglia activation. Coincident with NWL283 delivery for 8 days, stroke-injured mice exhibited improved functional outcomes that persisted following cessation of the drug. Therefore, we propose that NWL283 is a promising therapeutic warranting further investigation to enhance stroke recovery.

## 1. Introduction

Stroke is the third leading cause of death and disability worldwide, and the incidence of stroke continues to increase. Traditional therapies require intervention to take place within hours of stroke, and can be used in a very small subset of patients. Furthermore, while rehabilitation promotes neuroplasticity of the brain, there is incomplete recovery after stroke.

Following stroke, rapid necrotic cell death occurs at the ischemic core. In the perilesional region, referred to as the penumbra, neurons undergo apoptotic cell death as a result of oxidative stress, excitotoxicity and inflammation over a period of days to weeks [1]. Therapeutic interventions are aimed at enhancing the survival of cells in the penumbra due to the slower progression of cell death in this region. One approach is to block caspase-dependent activation of the apoptotic pathway. Caspases can be classified into: (i) proinflammatory caspases (caspases 1 and 11); (ii) initiator caspases (2, 8, 9 and 10); and effector caspases (3, 6 and 7) [2]. Upstream initiator caspases converge onto and activate downstream effector caspases that enzymatically cleave proteins and are responsible for initiating cell death. Currently, caspase inhibitors are being tested in clinical trials for the treatment of liver damage [1]. With regard to the central nervous system, there is some evidence that pan-caspase and specific caspase inhibitors can provide neuroprotection, but clinical trials to treat stroke have not been successful to date [2]. A major concern that impacts the use of caspase inhibitors for CNS repair is their off-target effects and inability to readily cross the blood–brain barrier (BBB) [3]. Development and use of novel, selective inhibitors that can readily cross the BBB are needed [4].

The adult brain contains a population of neural stem cells and their progeny; together, these are called neural precursor cells (NPCs). These rare, self-renewing and multipotent cells hold promise for promoting neural repair through cell replacement and enhancing neuroplasticity [5,6]. NPCs are found in a well-defined region lining the lateral ventricles in the forebrain. Under baseline conditions and following injury, NPCs undergo cell death in their periventricular niche (subventricular zone, SVZ) through a receptor-mediated apoptosis pathway [7,8]. An injury, such as stroke, can induce the resident NPCs to proliferate and migrate from the periventricular region of the forebrain to the site of stroke injury. However, the number of NPCs that migrate and survive in the cortex following stroke is insufficient to promote neural repair [9,10,11,12]. Therapeutics targeting NPC survival, such as cyclosporin A, have been shown to improve stroke outcomes [13,14,15]. Studies suggest that surviving NPCs can differentiate to replace dying neurons or provide trophic support after injury [11,16,17]. Hence, therapies that target downstream effector caspase-3 and caspase-7 have the potential to improve NPC survival in the brain, both in their periventricular niche as well as in the cortical stroke injury site.

Herein, we tested the efficacy of a propriety vinyl methyl sulfone inhibitor, NWL283, that selectively targets caspase-3/7 to promote cell survival. This proprietary peptidomimetic drug is an irreversible antagonist of the caspase-3/7 Cys163 catalytic active site. NWL283 promoted NPC survival in vitro and in vivo in a preclinical mouse model of cortical stroke. We demonstrate that administration of NWL283 immediately following stroke improves motor function and gait, reduces neuronal and NPC death at early time points post-stroke, enhances neurogenesis from NPCs and reduces microglial activation. Hence, the pro-survival drug NWL283 is neuroprotective, has neurogenic potential and improves functional outcomes after stroke.

## 2. Results

### 2.1. NWL283 Prevents Cell Death Following Stroke

Ischemic stroke triggers cellular events that lead to both apoptotic and necrotic neuronal cell death in a progressive manner. We asked if preventing stroke induced apoptotic cell death using a novel peptidomimetic drug that is an irreversible antagonist of the caspase-3/7 Cys163 catalytic active site would lead to improved outcomes following stroke. While previous selective caspase-3 inhibitors, such as zVAD-fmk, have been shown to be non-specific in binding caspase-3 with poor penetration into the brain [18], NWL283 is a highly water-soluble (400 mg/mL) caspase-3/7 specific inhibitor with a lipophilic and peptidic motif designed for active transport across the BBB. Testing in mice revealed that the half-life of the free form of NWL283 was ~2 h, while the half-life for the active (bound) form of NWL283 was estimated at ~3 days. This is similar to previous reports using NWL117 and NWL154, which are vinyl methyl sulfone inhibitors of caspase-6 (Pakavathkumar et al., 2017). The brain/plasma ratio of NWL283 was >3.0 due to active transport of NWL283 across the BBB. As a comparison, the half-life of the Merck drug, M867 was found to be around 40 min, with no detectable BBB penetration [19,20]. With regard to specificity, NWL283 showed a half maximal inhibitory concentration (IC50) of 8 nM against caspase-3 and 21 nM against caspase-7; with minimal inhibition against other caspases (Table S1).

**Figure 1:**
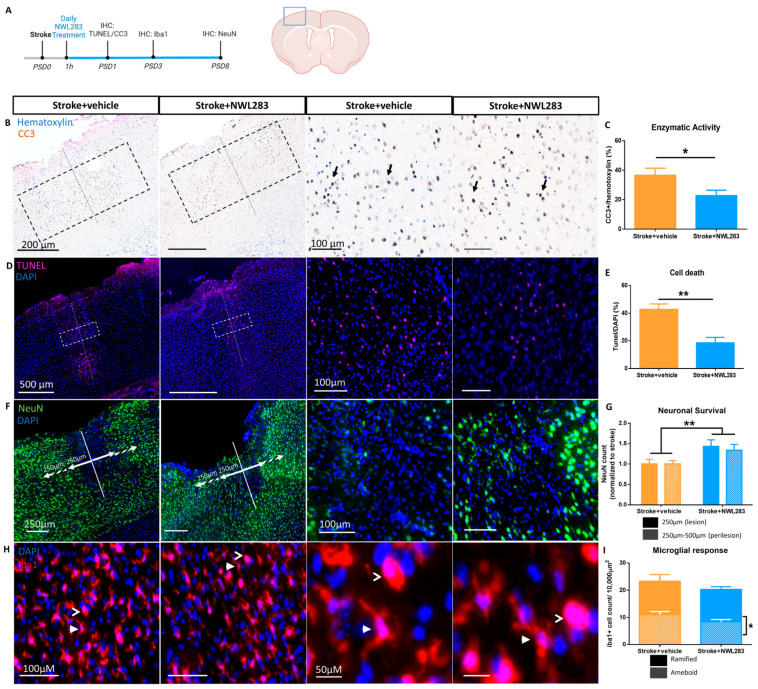
NWL283 administration leads to neuroprotection following stroke. (**A**) Schematic of experimental design. Box represents the region of cortical stroke on schematic coronal section. (**B**) Stroke+vehicle and stroke+NWL283 tissue sections stained for cleaved caspase-3 (CC3) and hematoxylin. Dashed line indicates the centre of the stroke lesion. CC3+ cells (arrows) were counted in the dashed box surrounding the stroke core. (**C**) CC3 expression was significantly reduced in NWL-treated mice following stroke. * *p* < 0.05, Student’s *t*-test. *n* = 7–9 mice/group; data represent mean ± SEM. (**D**) TUNEL+ cells were quantified in the dashed box within the stroked cortex. (**E**) Significantly fewer TUNEL+ cells were observed in NWL treated mice ** *p* < 0.01, Student’s *t*-test. Data represent mean ± SEM, *n* = 4 mice/group. (**F**,**G**) NeuN+ neurons were counted 250 µm medial and lateral to the stroke core (lesion) and in the perilesional area (250–500 µm from the stroke core). Stroke+vehicle mice had significantly fewer NeuN+ neurons in the lesion and perilesional area compared to stroke+NWL283 mice. * *p* < 0.05; Two-way ANOVA, Bonferroni post hoc. *n* = 6 mice per group; data represent mean ± SEM. (**H**) The number of Iba1+ microglia (arrows) were assessed based on their morphology and categorized as reactive microglia (extending <1 process from the cell body, ameboid; open arrow) or as homeostatic microglia (two or more processes, ramified; closed arrow). (**I**) Stroke+vehicle mice exhibited similar levels of ameboid and ramified microglia. NWL283 treatment reduced the number of reactive ameboid microglia compared to ramified microglia at PSD3. * *p* < 0.05; Student’s *t*-test. *n* = 3; mean ± SEM.

**Figure 2:**
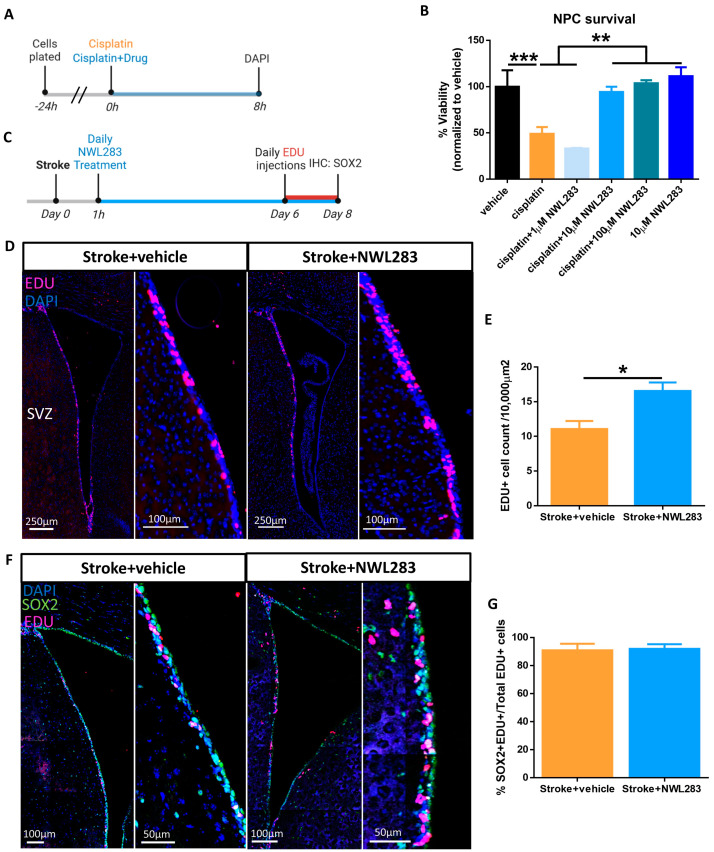
NWL283 prevents neural precursor cell death. (**A**) Schematic of cell culture plating and treatment time points. (**B**) Cisplatin led to a significant loss of NPCs at 8 h. In the presence of ≥10 µm NWL283 no NPC cell death was observed. ** *p* < 0.01, *** *p* < 0.001, One-way ANOVA, Tukey’s post hoc. *n* = 3 independent experiments; data represent mean ± SEM. (**C**) Schematic of experimental design. (**D**) Representative images of EDU labelled cells within the SVZ of stroke+vehicle- and stroke+NWL28-treated mice. (**E**) The number of EDU+ cells in the SVZ increased with NWL283 administration. * *p* < 0.05, Student’s *t*-test. *n*= 3–4 mice per group; data represent mean ± SEM. (**F**,**G**) Proliferating NPCs (EDU+SOX2+ cells) account for the vast majority of EDU+ cells (>90%) in both stroke+vehicle- and stroke+NWL283-treated mice. NWL283 promotes survival of endogenous NPCs post-stroke. *n* = 3–4 mice per group; data represent mean ± SEM.

We asked whether the administration of NWL283 would provide neuroprotection in a preclinical mouse model of stroke. In the first of a series of experiments, young adult male mice received an endothelin-1 stroke to the sensory motor cortex on day 0. This was followed by 21 mg/kg NWL283 injections beginning at 1 h post-surgery and continuing daily for up to 8 days (Figure 1A). Previous studies have shown that post-stroke day 1 (PSD1) represents a peak time for cell death in the ischemic core, as indicated by the presence of TUNEL labelling (a marker of cell death) and cleaved caspase-3 (a marker of apoptosis) [21,22]. Hence, we examined the expression of cleaved caspase-3 (CC3) and TUNEL at PSD1 in mice treated with NWL283 (stroke+NWL283) or vehicle (stroke+vehicle). The percentage of CC3+/Hematoxylin+ cells was significantly reduced in stroke+NWL283 compared to stroke+vehicle mice (stroke+vehicle = 36.62 ± 4.80% vs. stroke+NWL283 = 22.76 ± 3.73%) (*p* = 0.049) (Figure 1B,C). We also observed significantly fewer TUNEL+DAPI+ cells in the drug-treated mice (stroke+vehicle = 42.85 ± 3.88% vs. stroke+NWL283 = 18.54 ± 3.87%, TUNEL+DAPI+ cells) (*p* = 0.004) (Figure 2D,E). Similarly, there was a significant reduction in TUNEL+NeuN+ cells in the drug-treated mice (stroke+vehicle = 16.85 ± 3.01% vs. stroke+NWL283 = 6.73 ± 0.10%, TUNEL+NeuN+/DAPI+ cells) (*p* = 0.036) (Figure S1). Together, these results indicate that NWL283 administration leads to significantly reduced cell death at early times post-stroke.

### 2.2. NWL283 Promotes Neuronal Survival Post-Stroke

Neuronal cell death occurs rapidly in the ischemic core and continues in the penumbra (perilesional area), causing a significant loss of neurons at one week after stroke [21,23]. To determine whether neuronal survival was improved with administration of NWL283, mice received stroke and daily injections of NWL283 and were sacrificed on PSD8 for tissue analysis. The number of NeuN+ neurons was assessed in two regions: (i) within 250 µm of the ET-1 injection site (core), and (ii) 250–500 µm (perilesion, extending medial and lateral to the core) (Figure 1F). A greater number of NeuN+ cells was observed in the stroke+NWL283 mice across these two regions (30 ± 0.88% increase, *p* = 0.008) (Figure 1G). Hence, NWL283 reduced neuronal cell death following stroke.

### 2.3. NWL283 Reduced Microglia Activation Following Stroke

We asked if the neuroprotective effects of NWL283 were correlated with a reduction in the microglial activation, which can serve as an indicator of neuroinflammation [24,25]. We examined the number and morphology of the microglia in stroked brains, with and without NWL283 treatment at PSD3, which has been shown to be a time of maximal reactivity [26,27,28]. The number of ramified (resting) and ameboid (reactive) Iba1+ microglia was counted at PSD3 within 250 µm of the stroke lesion site (Figure 1H). We found no difference in the total numbers of microglia between stroke+vehicle- and stroke+NWL283-treated brains; however, the number of reactive ameboid microglia was significantly reduced relative to resting ramified microglia in the NWL283 treated mice (*p* = 0.039) (Figure 1I). This suggests that the microenvironment is altered with NWL283 treatment.

### 2.4. NWL283 Enhances Neural Precursor Cell Survival

Endogenous NPCs in the periventricular stem cell niche (SVZ) have been shown to migrate to the stroke lesion site within days following stroke [9,12]. However, the vast majority of NPCs do not survive and integrate in the parenchyma but rather die en route or within the lesion site [9,29,30,31]. We first asked whether NWL283 was able to improve NPC survival using an in vitro assay. Neurospheres (colonies of NPCs) were isolated from the SVZ of adult mice (no stroke) then dissociated and plated as single cells in the presence or absence of cisplatin: a DNA chelating agent that induces apoptosis [32]. We performed a dose–response curve and determined that 20 µM cisplatin concentration induced apoptosis in approximately 70% of NPCs at 8 h after exposure (Figure S2). Next, to assess the effects of NWL283 on cell survival, NPCs were plated in vehicle, 20 µM cisplatin, and 20 µM cisplatin+NWL283 (1 µM, 10 µM and 100 µM) and cell viability was assessed at 8 h post-plating by counting the numbers of DAPI+ cells (Figure 2A). With the addition of NWL283, cell survival was significantly improved compared to cisplatin treatment (cisplatin = 49.09 ± 7.28 vs. cisplatin + 10 µM NWL283 = 94.29 ± 5.62, DAPI+ cells) (*p* = 0.0014) and was not significantly different from control (vehicle) (*p* = 0.986) (Figure 2B). Importantly, NWL283 treatment alone (i.e., no cisplatin to induce cell death) did not increase the number of NPCs. This suggests that NPC proliferation did not account for the number of DAPI+ cells in cisplatin+NWL283-treated cultures (Figure 2B). Hence, NWL283 was able to enhance NPC survival in vitro in the presence of an apoptosis-inducing drug.

### 2.5. NWL283 Promotes Survival of Endogenous NPCs Post-Stroke

Based on the pro-survival effect on NPCs observed with NWL283 in vitro, we predicted that in vivo administration of NWL283 would enhance the survival of endogenous NPCs following stroke. Endogenous NPCs proliferate in the SVZ and express Nestin and SOX2. The majority of newborn NPCs die under baseline conditions [33] with a subpopulation migrating to the olfactory bulb and generating interneurons [34,35]. To ask whether NWL283 treatment enhanced the survival of endogenous NPCs in the SVZ post-stroke, cohorts of mice received stroke+vehicle or stroke+NWL283 and were injected with the proliferation marker EDU for 2 days prior to tissue harvesting on PSD8 (Figure 2C). We found that the number of EDU+ cells were significantly increased following stroke+NWL283 treatment compared to stroke+vehicle (stroke+vehicle = 11.06 ± 1.16 vs. stroke+NWL283 = 16.54 ± 1.23, EDU+ cells per 10,000 µm^2^) (*p* = 0.026) (Figure 2D,E). Further, the vast majority of EDU+ cells within the SVZ expressed SOX2+ in both treatment groups (>90%) (Figure 2F,G). We did not find a concomitant increase in the number of EDU+ cells within the cortex of stroke+NWL283-treated mice (Figure S3). Hence, NWL283 administration resulted in an expansion of the NPC pool in the SVZ at PSD8.

**Figure 3:**
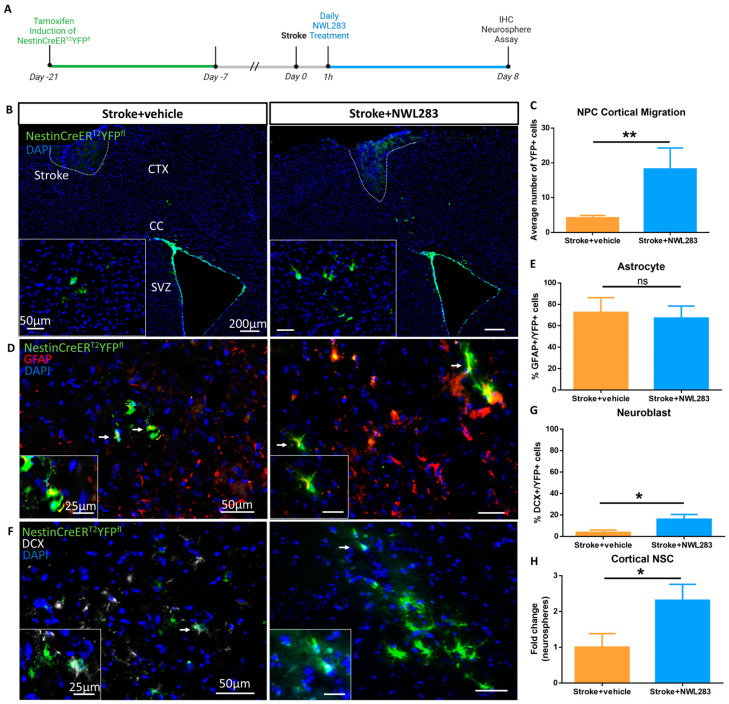
NWL283 enhances NPC migration to the stroke-injured cortex. (**A**) Schematic of experimental paradigm in NestinCreER^T2^YFP^fl^ mice. (**B**) YFP+ NPCs are observed in the SVZ and in the stroke-injured cortex and corpus callosum. Insets are higher magnification images of YFP+ cells. Dashed line represents cortical lesion. (**C**) NWL283-treated mice have significantly more YFP+ SVZ-derived cells in the stroke-injured hemisphere compared to stroke+vehicle mice. ** *p* < 0.01, Student’s *t*-test; *n* = 4–6 mice per group; data represent mean ± SEM. (**D**) YFP+ cells expressed GFAP following stroke (arrows). Insets are higher-magnification YFP+GFAP+ cells. (**E**) Quantification of YFP+GFAP+ cells within the injured cortex revealed no significant difference between treatment groups. ns = not significant. Student’s *t*-test; *n* = 4–6 mice per group; data represent mean ± SEM. (**F**) YFP+DCX+ cells were examined in the stroke-injured cortex and corpus callosum (arrows). (**G**) Quantification reveals a small but significant increase in the subpopulation of YFP+ cells co-expressing DCX+ in the stroke-injured cortex of stroke+NWL283 mice compared to the stroke+vehicle mice. * *p* < 0.05, Student’s *t*-test. *n* = 4–6 mice per group; data represent mean ± SEM. (**H**) Significantly more neurospheres were isolated from the stroke-injured cortex of mice treated with NWL283 compared to stroke+vehicle. * *p* < 0.05, Student’s *t*-test. *n* = 6 mice per group; data represents mean fold change ± SEM.

Under baseline conditions, NPCs are confined to the SVZ (Figure S4). Following stroke, NPCs migrate through the corpus callosum and into the ipsilateral stroked cortex towards the injury site [9,12]. Previous work has shown that migrating NPCs do not survive at the stroke injury site [9]. To determine if NWL283 enhanced the migration and/or survival of NPCs in the stroke-injured cortex, we performed lineage tracking in NestinCreER^T2^YFP^fl^ reporter mice that enabled permanent labelling of Nestin+ NPCs in the SVZ prior to stroke and subsequent tracking of YFP+ NPCs and their differentiated progeny. Mice received TAM chow to label NPCs (YFP+) and were divided into stroke+vehicle or stroke+NWL283 groups (Figure 3A). The numbers of YFP+ NPCs (derived from the SVZ) were counted within the ipsilateral stroke-injured hemisphere on PSD8. As shown in Figure 3B, YFP+ cells were found in the corpus callosum and the stroke-injured cortex. Quantification revealed a significant 4.3-fold increase in YFP+ cells in the parenchyma of stroke+NWL283-treated mice compared to stroke+vehicle-treated mice (stroke+vehicle = 4.22 ± 0.66 vs. stroke+NWL283 = 18.25 ± 6.04, YFP+ cells) (*p* = 0.013) (Figure 3C) on PSD8. Hence, NWL283 increased the number of SVZ-derived NPCs in the parenchyma of the stroke-injured hemisphere.

To determine whether the NPC-derived YFP+ cells were differentiating, immunohistochemistry was performed for GFAP (astrocyte marker), DCX (neuroblast, immature neuronal marker) and NeuN (mature neuronal marker) (Figure 3D–G). There was no significant difference in the number of NPC-derived YFP+ cells that differentiated into GFAP+ astrocytes following NWL283 treatment compared to stroke+vehicle mice (*p* = 0.772) (Figure 3E). However, there was a significant increase in the numbers of YFP+ cells that differentiated into DCX+ neuroblasts in the stroke+NWL283 group compared to stroke+vehicle (stroke+vehicle = 3.78 ± 2.22% vs. stroke+NWL283 = 16.03 ± 4.42%) (*p* = 0.038) (Figure 3G). Notably, we observed increased numbers of YFP+ cells in the cortex of stroke+NWL283 mice compared to stroke+vehicle mice (Figure 3B,C). To determine whether these YFP+ SVZ-derived cells differentiated and expressed the mature neuronal marker NeuN+, the number of YFP+NeuN+ cell was assessed. A very small subpopulation of YFP+NeuN+ cells was observed in the NWL-treated (3%) and vehicle-treated mice (4%), indicating that the majority of SVZ-derived, migrating cells did not differentiate into mature neurons by PSD8. Finally, based on previous reports demonstrating neural stem cell migration to the stroke-injured cortex [12,14], we performed the neurosphere assay on cortical tissue from stroke-injured mice. Stroke+NWL283 mice had a significant 2.3-fold increase in the number of cortical-derived neurospheres compared to stroke+vehicle mice (stroke+vehicle = 1.00 ± 0.38 vs. stroke+NWL283 = 2.31 ± 0.44, neurospheres per 5000 cells) (*p* = 0.036) (Figure 3H). Taken together, these findings reveal that NWL283 increased the number of neural stem cells and progenitors within the stroke-injured cortex, affecting neurogenesis.

### 2.6. NWL283 Improves Motor Deficits after Stroke

**Figure 4:**
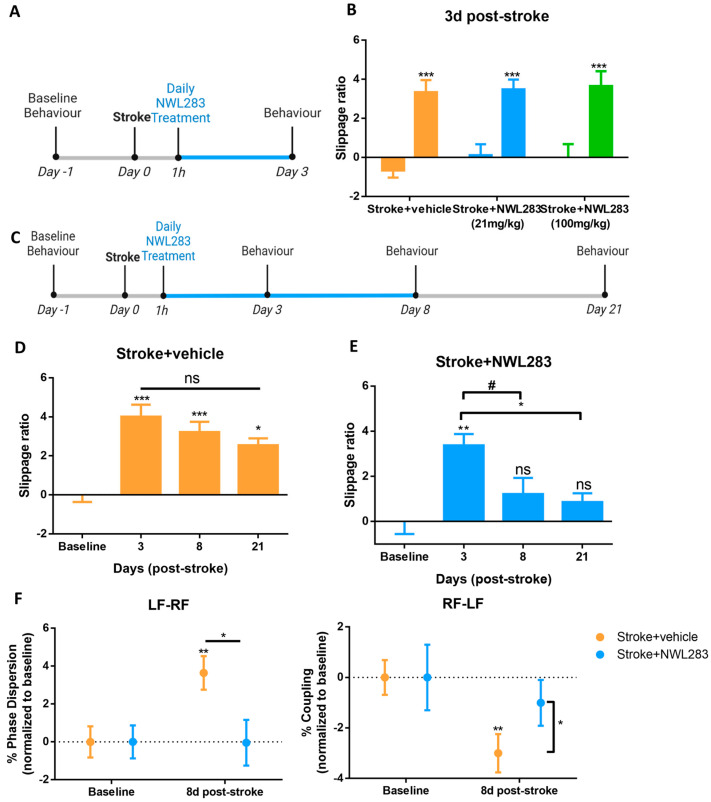
NWL283 improves functional outcomes following stroke. (**A**) Schematic of experimental timeline. (**B**) Stroke+vehicle and stroke+NWL283 mice had increased slippage ratios at PSD3, irrespective of NWL283 dose. *** *p* < 0.001, two-way ANOVA, Sidak post hoc. Stroke+vehicle; *n* = 7 mice Stroke+NWL283 (21 mg/kg); *n* = 8 mice, Stroke+NWL283 (100 mg/kg); *n* = 5 mice. Data represent mean ± SEM. (**C**) Schematic of experimental design. (**D**) Stroke+vehicle mice have significantly greater foot-fault slippage ratios compared to baseline performance at all times examined and not significantly differ over time. ns = not significant, * *p* < 0.05, ** *p* < 0.01, *** *p* < 0.001, one-way ANOVA, Dunnett post hoc. PSD3, PSD8; *n* = 14 mice, PSD21; *n* = 5 mice. Data represent mean ± SEM. (**E**) Stroke+NWL283-treated mice performed similar to baseline levels at PSD8 and PSD21, with a time dependent reduction in slippage ratios at PSD8 and PSD21, compared to PSD3. * *p* < 0.05, ** *p* < 0.01, one-way ANOVA, Dunnett post hoc. # *p* < 0.05, Student’s *t*-test. PSD3, PSD8; *n* = 8 mice, PSD21; *n* = 5 mice. Data represent mean ± SEM. (**F**) Stroke+vehicle mice display increased phase dispersion and decreased coupling of the stroke-affected (**left**) and unaffected (**right**) forepaws when compared to baseline and relative to NWL283-treated mice. * *p* < 0.05, ** *p* < 0.01, two-way ANOVA, Bonferroni post hoc. *n* = 7–10 mice per group; mean ± SEM.

Given the enhanced cell survival with NWL283 treatment following stroke, we next sought to determine whether NWL283-treated mice had improved functional outcomes either by (i) preventing impairments early post-stroke or (ii) by enhancing recovery over time. To assess whether NWL prevented motor impairments, we looked at early times post-stroke following treatment. Mice received vehicle or NWL283 at the time of stroke, and then once daily for 3 days at 21 mg/kg (as was used for the cellular analysis) or 100 mg/kg concentration (Figure 4A). On PSD3, mice were tested using the foot-fault task to assess gross motor function. As shown in Figure 4B, all stroke-injured mice displayed significant motor impairments at PSD3 (increased percent slippage compared to baseline performance), irrespective of NWL283 dose. Further, the magnitude of the deficit was similar across all groups. Hence, NWL283 did not reduce the acute motor impairments that resulted following ET-1 cortical stroke.

To determine whether the administration of NWL283 resulted in functional recovery at later times post-stroke, mice were treated daily with the low-dose 21 mg/kg NWL283 until PSD8 and assessed on the foot-fault task and gait analysis at baseline, PSD3, PSD8 and PSD21 (Figure 4C). Stroke+vehicle mice continued to display significant increases in the percent slippage up to PSD21 relative to their own baseline (4.01 ± 0.62, 3.22 ± 0.52, 2.55 ± 0.35 percent slippage on PSD3, 8 and 21, respectively; PSD3, *p* < 0.0001; PSD8, *p* = 0.0001; PSD21, *p* = 0.03) (Figure 4D). Strikingly, stroke+NWL283 mice displayed improved functional outcomes, with the percent slippage returning to baseline values at PSD8 (1.21 ± 0.72 percent slippage; *p* = 0.44) and this was maintained to PSD21 (0.86 ± 0.39 percent slippage; *p* = 0.78) (Figure 4E). Furthermore, stroke+NWL283 mice significantly improved over time, compared to stroke+vehicle mice (effect of treatment, *p* = 0.017) (Figure 4E). These findings reveal that a longer duration of NWL283 treatment led to improved motor function after stroke.

We further evaluated motor function using CatWalk gait analyses to monitor gait. Similar to previous studies, we observed increased phase dispersion (irregular placement of forepaws) and decreased coupling (asynchronous paw placement) in our stroke+vehicle mice at PSD8 (Figure 4F) [36]. Notably, stroke+NWL283 mice displayed phase dispersion and coupling that resembled their baseline values and were improved compared to stroke+vehicle (phase dispersion: stroke+vehicle = 3.64 ± 0.89% vs. stroke+NWL283 = −0.041 ± 1.21%; *p* = 0.048) (coupling: stroke+vehicle = −3.0 ± 0.76% vs. stroke+NWL283 = −1.0 ± 0.91%; *p* = 0.028) (Figure 4F). Hence, gait analysis demonstrated that abrogated temporal paw patterns induced by stroke were improved with NWL283 treatment for 8 days post-stroke.

## 3. Discussion

Ischemic injury induces neuronal loss, resulting in irreversible damage that is exacerbated by the inflammatory response [37,38,39]. While the majority of neurons are rapidly lost due to necrosis, neurons continue to undergo apoptosis for weeks following the insult [23,38]. Pharmacological blockers of apoptotic signalling, such as caspase-3 inhibitors, have been shown to improve cellular and functional outcomes following neural injury. However, a poor understanding of their pharmacokinetics renders them unsafe for clinical use [40]. Herein, we have explored the efficacy of a novel caspase-3 and -7 inhibitor, NWL283, in a preclinical mouse model of ischemic stroke. We have demonstrated that daily systemic administration of NWL283 reduces neuronal cell death and microglia activation soon after stroke. Interestingly, these neuroprotective effects and immunomodulation were not sufficient to reduce the functional impairments that result from the ischemic insult. The pro-survival effects of NWL283 were further demonstrated with enhanced endogenous NPC survival and migration to the stroke injury site. Most interestingly, we reveal that systemic delivery of NWL283 for 8 days post-stroke leads to improved motor outcomes that are sustained upon cessation of the drug treatment. Hence, our results show that the pleiotropic effects of NWL283 treatment on neurons, NPCs and microglia cumulatively recover lost motor function and maintain recovery post-treatment.

NWL283 is an irreversible blocker for caspase-3 and -7, which are downstream effector caspases initiating proteolytic cleavage of proteins and inducing cell death. While NWL283 reduced cleaved caspase-3 levels in vivo, the drug did not cause a complete absence of enzymatic expression similar to other peptidic vinyl methyl sulfone caspase inhibitors [4]. It is plausible that despite the drug rendering the enzyme non-functional, the presence of the enzyme within cells persists due to a slow degradation process [41]. A decrease in TUNEL labelling indicates that the drug is indeed reducing cell death. Although caspase-3-mediated apoptosis is a significant mode of cell death following stroke, other cell death pathways involving necroptosis is also targeted by NWL283 [42]. Therefore, reductions in cleaved caspase-3 expression and TUNEL-labelled cells positively correlated with increased neuronal survival after NWL283 treatment and a concomitant improvement in motor function.

The canonical apoptosis pathway is mediated through caspase-3 activity. Consistent with previous reports, we found that the administration of a caspase-3 inhibitor reduces neuronal death [42,43]. Similarly, endogenous NPCs that migrate to the injury site following stroke have been shown to undergo caspase-3-mediated cell death and, importantly, NPC cell death can be prevented using a pan-caspase inhibitor such as zVAD-fmk [44]. Excitingly, we found that the use of a selective caspase-3 and -7 inhibitor, NWL283, prevented NPC death following stroke and this resulted in an increase in the number of NPCs and their progeny within the ischemic cortex. Our study suggests that the survival of migrating NPC and their progeny can be improved by selectively targeting the canonical caspase-3 mediated apoptotic pathway in these cells. Therefore, we showed that NWL283 is protective against cell death, leading to increased survival of neurons, NPCs and their progeny.

Previous apoptosis blockers have shown promise in preventing neurodegeneration. However, the concentrations required to achieve the desired effect resulted in extrapyramidal adverse effects. Selective caspase inhibitors that can efficiently cross the BBB are required to exert neuroprotective effects in vivo [4]. NWL283 has a significantly lower IC50 and higher BBB permeability than conventional caspase-3 inhibitors [45]. Previous clinical trials using caspase-3 inhibitor drugs have not been fruitful due to lack of specificity or high doses [40]. Previous studies have shown that low concentrations of vinyl sulfone inhibitors do not contribute to adverse effects in mice [4,46]. A low NWL283 concentration was used to test the therapeutic effects during an acute stroke, demonstrating a lack of in vivo NPC cell death and unaffected mouse survival. Our results demonstrated that NWL283 can inhibit caspase-3 at physiologically low concentrations in mice.

We tested NWL283 in a rodent model of stroke and found that the length of treatment was important for motor outcomes. Indeed, 3 days of treatment is insufficient to prevent functional deficits; this is potentially due to the necrotic processes that ensue immediately after stroke [47]. However, a longer duration of treatment (8 days) improved motor outcomes in more than one functional assay. This finding is consistent with the fact that cells undergo apoptosis over the course of days to weeks post-stroke and thus can be protected with anti-apoptotic interventions over an extended time. Previous studies have also found that a 24 h delayed treatment using caspase-3 inhibitors causes less motor recovery after injury; this highlights the importance of assessing the time window for therapeutic benefit [48,49]. Therefore, treatment using caspase-3 inhibitors is optimal early on post-stroke and for a longer duration.

Interestingly, NWL283 resulted in an expansion of the SVZ proliferating pool, NPC migration and neurogenesis within the injured cortex. At the time when functional recovery was observed, the NPCs in the stroke-injured cortex had differentiated primarily into GFAP+ astrocytes [12,50,51]. Concomitantly with enhanced NPC migration, the administration of NWL283 promoted neurogenesis from the NPC-derived neuroblasts. While these neuroblasts are immature, studies have shown that increasing the number of neuroblasts alone can improve stroke outcomes [52,53,54]. Astrocytes can exhibit a pro-inflammatory or an anti-inflammatory state. Astrocytes have been shown to release anti-inflammatory and neuroprotective cytokines, such as TGFβ1, during the subacute phase of stroke [55]. Therefore, migrating NPCs that mature into astrocytes, in addition to endogenous NPCs, can provide factors that support neuroplasticity within the ischemic cortex [14,50,56,57,58,59,60]. To further promote a permissive microenvironment for neuronal repair, NWL283 administration reduced immune activation. NWL283 can reduce immune activation either indirectly by reducing neuronal cell death, causing an attenuated immune response; or directly by inhibiting caspase-3 dependent microglial activity [61,62,63]. This additional effect of NWL283 on inhibiting microglial activation may prevent cytokine-induced cell death. Hence, NWL283 can modulate the microenvironment after stroke, which has the potential to be more permissive to neuroplasticity.

In conclusion, NWL283 is a promising drug that selectively inhibits caspase-3 and -7 activity. NWL283 can prevent cell death, alter the microenvironment and improve functional outcomes after stroke. The improved stroke outcomes observed with NWL283 treatment may also be attributed to the enhanced activation of endogenous NPCs and their progeny. Whether NWL283 affects NPC survival through a canonical caspase-3-mediated apoptosis pathway remains to be determined. Future experiments to determine the therapeutic potential of NWL283 in other degenerative diseases would be of interest.

## 4. Materials and Methods

### 4.1. Animals

Male C57BL/6 mice aged 6–8 weeks were used for behaviour and imaging. Animals were housed with ad libitum food and water, in a room with a 12 h light/dark cycle. Nestin-CreERT2 mice [64] were crossed with YFPfl mice [65] to generate NestinCreERT2YFPfl mice [12]. All experiments were conducted in accordance with the University of Toronto Faculty of Medicine and Pharmacy Animal Care Committee and with Canadian Council on Animal Care guidelines for the care and use of animals.

For NWL283 half-life and blood–brain barrier penetration studies, male and female C57BL/6 mice aged 6–8 weeks were used. All experiments were conducted in accordance with the University of Montreal Animal Care Committee and with Canadian Council on Animal Care guidelines for the care and use of animals.

### 4.2. Caspase Inhibition Assays

The IC50 of NWL283 for each of Caspase 1–10 was determined using an enzymatic inhibition assay (Caspase-1 to -10 Inhibitor Drug Screening Kit, Biovision, Waltham, MA, USA) as per the manufacturer’s instructions (Table S1). Briefly, 50 µL of NWL283 in dH_2_O was added to 5 µL of active caspases 1–10, 45 µL of reaction buffer (containing 10 mM DTT) and 50 µM LEHD-AFC substrate. The sample was incubated at 37 °C for 1 h and read using a fluorescence plate reader at 400 nm excitation and 505 nm emission filters.

### 4.3. Half-Life and Blood–Brain Barrier (BBB) Penetration Studies

To assess the half-life and BBB penetration, 30 mg/kg of NWL283 was injected once intraperitoneally (IP) into 30 mice, and the mice were sacrificed at different time points. The blood and organs were harvested and processed for liquid chromatography analysis of NWL283 levels.

### 4.4. Stroke and Drug Administration

Mice were anaesthetized using isoflurane and injected with 5 mg/kg meloxicam. A hole was drilled into the skull above the M1 and M2 motor cortex using bregma coordinates AP: +0.6, ML: +2.2, DV: −1.0. A 2.5 μL Hamilton Syringe with a 26 gauge, 0.375″ needle (Hamilton, Reno, NV, USA) was used to inject 1 μL of 800 µmol Endothelin-1 (ET-1) (MilliporeSigma, 05-23-3800, Oakville, ON, Canada) at a rate of 0.1 μL/min. The needle was withdrawn 10 min after injection. At 1 hour post-surgery, mice received an intraperitoneal (i.p.) injection of NWL283 (New World Laboratories, Laval, QC, Canada) in sterile saline at a dose of 21 mg/kg or 100 mg/kg; or sterile saline only (vehicle control). Mice received one injection daily for 3- or 8-days post-stroke.

### 4.5. EDU Treatment

Mice were injected with 50 mg/kg EDU (Invitrogen, E10187, Waltham, MA, USA) once daily (i.p.) for two days, PSD6 and PSD7, prior to sacrifice on PSD8. Three to four animals per group were used for immunostaining.

### 4.6. Tamoxifen

NestinCreER^T2^YFP^fl^ mice were fed tamoxifen chow (Envigo, Teklad 0.5% TAM and 5% sucrose, Indianapolis, IN, USA) ad libitum for 2 weeks followed by a 1-week chase period, when the animals received regular chow (Envigo, Teklad 2019, Indianapolis, IN, USA). Four to six mice were used for YFP+, YFP+GFAP+ and YFP+DCX+ cell counts.

### 4.7. Tissue Processing

Mice were euthanized with 250 mg/kg Avertin (Sigma-Aldrich, St. Louis, MO, USA) and intracardially perfused with ice-cold PBS using peristaltic pump (Cole-Palmer Masterflex, 7518-10, Quebec, QC, Canada) at a rate of 20 mL/min for 2 min, followed by 30 mL of 4% paraformaldehyde (Sigma-Aldrich, St. Louis, MO, USA) at a rate of 15 mL/min. Brains were post-fixed at 4 °C in 4% paraformaldehyde and either stored in 30% sucrose for cryosectioning or processed for paraffin embedding. Brains were frozen in OCT (Thermo Fisher Scientific, Waltham, MA, USA), cryosectioned (−20 °C) at 20 µm sections onto SuperfrostPlus slides (Thermo Fisher Scientific, Waltham, MA, USA) and stored at −20 °C until staining was performed. Sections were collected at every 160 µm interval. Paraffin-embedded brains were mounted onto slides as 5 µm sections and processed for staining.

### 4.8. Immunohistochemistry

Sections were thawed, washed once with PBS and blocked using 4% normal goat serum and 0.3% Triton X-100 (BioShop, TRX777, Burlington, ON, Canada) in PBS for 1 h at room temperature. Sections were incubated with primary antibodies 1:200 anti-NeuN (Millipore Sigma, ABN78, Oakville, ON, Canada), 1:500 anti-Iba1 (Wako, 019-19741, Osaka, Japan), 1:500 anti-GFP (Aves Labs, GFP-1010, Davis, CA, USA), 1:250 anti-DCX (Abcam, ab18723, Cambridge, UK) and 1:400 anti-GFAP (Sigma-Aldrich, G3893, St. Louis, MO, USA) in blocking solution overnight at 4 °C. Three PBS washes were performed, followed by incubation with secondary antibodies; 1:1000 Alexafluor488 and 568 (Invitrogen, Waltham, MA, USA) at room temperature for 1 h. After three PBS washes, sections were mounted using Vectashield with DAPI (VectorLabs, H-1200, Newark, CA, USA). Sections from a minimum of three animals were immunostained for cell counts.

Paraffin-embedded sections were dewaxed in xylene for 5 min and rehydrated for 5 min each in 100% EtOH, 95% EtOH and 70% EtOH. Peroxidase activity was blocked with 3% H_2_O_2_ in PBS for 15 min and washed with water for 5 min. Antigen retrieval was performed with heat-induced epitope retrieval in citrate buffer pH 6.0 for 10 min and rinsed with water. Tissue was blocked with 3% H_2_O_2_ in PBS for 10 min and protein block serum-free (Dako, X0909, Glostrup, Denmark) for 10 min. Sections were incubated with primary antibodies 1:200 anti-cleaved caspase-3 (Cell Signalling, 9661, Danvers, MA, USA) at room temperature for 30 min. Sections were triple-washed with PBS-Tween, incubated with 1:200 Anti-Rabbit Ig antibody (VectorLabs, BA-1000, Newark, CA, USA) at room temperature for 30 min, then incubated with 1:50 ABC detection system (Vector Labs, PK-6100, Newark, CA, USA) for 30 min and with DAB (Abcam, ab64238, Cambridge, UK) for 10 min. DAB-stained sections were dehydrated through a series of graded alcohol, which has been cleared in xylene prior to mounting. Seven to nine mice were labelled for cleaved caspase-3.

### 4.9. EDU Staining

Cryosectioned tissue underwent antigen retrieval with citrate buffer (pH 6.0) at 95 °C for 15 min. Sections were permeabilized and blocked in 4% NGS and 0.3% Triton X-100. Sections were stained with the Click-iT reaction cocktail, as indicated in the Click-iT EDU kit (Invitrogen, C10424, Waltham, MA, USA). Samples were washed three times with PBS and mounted using Vectashield with DAPI (VectorLabs, H-1200, Newark, CA, USA). Sections from a minimum of three animals were stained for cell counts.

### 4.10. TUNEL Staining

Cryosectioned tissues were processed according to the manufacturer’s directions (Thermo Fisher Scientific, C10619, Waltham, MA, USA). Briefly, sections were incubated with proteinase K solution for 15 min followed by a PBS rinse for 5 min. Sections were fixed in 4% paraformaldehyde for 15 min at 37 °C and washed twice with PBS for 5 min. Subsequently, TdT Reaction buffer was applied for 10 min at 37 °C followed by TdT reaction mixture (reaction buffer, EdUTP and TdT enzyme) for 1 h at 37 °C. After a single rinse in deionized water, sections were blocked and permeabilized using 3% BSA and 0.3% Triton X-100 in PBS for 5 min. Sections were rinsed with PBS and incubated with TUNEL reaction cocktail (TUNEL Reaction Buffer Additive, Copper Protectant and Alexa Fluor647 picolyl azide) for 30 min at 37 °C. Following three PBS washes, slides were mounted using Vectashield with DAPI (Vector, H-1200, Newark, CA, USA). Following TUNEL labelling, some sections were blocked using 4% normal goat serum and 0.3% Triton X-100 (BioShop, TRX777, Burlington, ON, Canada) in PBS for 1 h at room temperature and incubated with primary antibody 1:200 anti-NeuN (MilliporeSigma, ABN78, Oakville, ON, Canada) in blocking solution overnight at 4 °C. Three PBS washes were performed, and this was followed by incubation with secondary antibodies; 1:1000 Alexafluor488 (Invitrogen, Waltham, MA, USA) at room temperature for 1 h. After three PBS washes, sections were mounted using Vectashield with DAPI (Vector, H-1200, Newark, CA, USA). Sections from a minimum of three animals were immunostained for cell counts.

### 4.11. Tissue Analysis

Images were acquired on an Axio Observer Zeiss microscope and an Axioscan Slide Scanner (Zeiss, Jena, Germany) at 10× and 20× magnification for counting. Colocalized antibody staining with DAPI was counted using QuPath v0.1.2 software’s automated detection and subsequent manual verification by a blinded observer. For NeuN counts, four 250 µm × 250 µm ROI’S per section were counted from within and surrounding the lesion site counted from a minimum of two sections. Cleaved caspase-3 counts were manually counted within a 200 µm × 400 µm region in the stroke lesion and averaged from three brain sections. To quantify the numbers of YFP-positive cells in NestinCreERT2YFPfl mouse brain, the total numbers of YFP+ cells were counted within the ipsilateral cortex dorsal to the corpus callosum between crossing of the anterior commissure to the crossing of the corpus callosum across three sections. Microglia with one process or less were counted as ameboid, and two or more processes were counted as ramified. Microglia counts were performed within three 250 µm × 250 µm ROI’S per section situated within the lesion site, across three sections. EDU counts were performed using four 100 µm × 200 µm ROI’S per section within the SVZ and one 625 µm × 625 µm ROI per section within the cortex, across three sections. TUNEL counts were performed within a 560 µm × 350 µm ROI per section within the lesion site, across three sections. All counts were represented as per µm or mm area.

### 4.12. Neurosphere Assay

Six mice per group were sacrificed using isoflurane. The brains were removed, placed in a brain matrix on ice and 3 to 4, 2 mm sections (AP + 0.5 to AP − 1.5) were floated in a 15 mm petri dish (Thermo Fisher Scientific, 848317, Waltham, MA, USA) containing ice cold artificial cerebrospinal fluid (aCSF). A dissection knife (Fine Science Tools 10056-12, Foster City, CA, USA) was used to dissect an area approximately 1.3 µm × 2 µm in size around the stroke lesion, avoiding the corpus callosum. The tissue was kept on ice and processed as previously described [13,66]. Briefly, samples were enzymatically digested at 37 °C for 25 min using 1.33 mg/mL trypsin (Sigma-Aldrich, T1005, St. Louis, MO, USA), 0.76 mg/mL hyaluronidase (Sigma-Aldrich, H6254, St. Louis, MO, USA), and 0.13 mg/mL kynurenic acid (Sigma-Aldrich, K3375, St. Louis, MO, USA) in aCSF. Tissue was centrifuged at 1500 rpm for 5 min, the supernatant was removed, and the pellet was resuspended by trituration in 0.67 mg/mL ovomucoid (Worthington Biochemical, LS003086, Lakewood, NJ, USA). Samples were centrifuged at 1500 rpm for 5 min, supernatant was removed, and samples were resuspended by 40× trituration in ice cold 23% Percoll Plus (Thermo Fisher Scientific, 17544502, Waltham, MA, USA) in 1 × PBS. Samples were centrifuged at 1800 rpm for 5 min and the supernatant was gently discarded without disruption of the separation layers. Pellets were resuspended by 5× trituration in serum-free medium (SFM) containing 10× Dulbecco’s modified Eagle’s medium/F12, 30% glucose, 7.5% NaHCO3, 1 M Hepes, l-glutamine, hormone mix, and penicillin and streptomycin. Samples were centrifuged at 1500 rpm for 5 min, the supernatant was removed, and the pellet were resuspended by 40× trituration in SFM containing 20 ng/µL epidermal growth factor (GIBCO, PMG8041, Waltham, MA, USA), 10 ng/µL basic fibroblast growth factor (GIBCO, PHG0266, Waltham, MA, USA) and 2 µg/mL heparin (Sigma-Aldrich, H3149, St. Louis, MO, USA). Cells were counted using Trypan Blue and a hemocytometer, then plated at a density of 10 cells/µL. After 7 days of incubation at 37 °C with 5% CO_2_, NPC spheres greater than 80 µm were counted using a light microscope.

### 4.13. In Vitro Cell Viability

NSC neurospheres were obtained from control (injured) C57BL/6 periventricular dissections, as described above. Neurospheres were collected and mechanically disassociated into single cells and plated in 96-well plates coated with 4% Matrigel (Corning 354234, Corning, NY, USA) at a density of 20,000 cells per well in 100 µL of SFM with 20 ng/µL epidermal growth factor (GIBCO, PMG8041, Waltham, MA, USA), 10 ng/µL basic fibroblast growth factor (GIBCO, PHG0266, Waltham, MA, USA) and 2 µg/mL heparin (Sigma-Aldrich, H3149, St. Louis, MO, USA) (referred to as growth factors). One day later, the media was removed and replaced with fresh SFM, growth factors, 20 µM cisplatin (Sigma-Aldrich, P4394, St. Louis, MO, USA) and 10 µM NWL283 (New World Laboratories, Laval, QC, Canada) in sterile PBS. The vehicle group received media containing SFM, growth factors and PBS.

To assess cell viability at 2 h and 8 h, cells were fixed with 4% PFA for 15 min and washed three times for 5 min in PBS. Cells were incubated for 10 min with 1:10,000 DAPI (Invitrogen, D130, Waltham, MA, USA), then washed three times for 5 min in PBS and imaged on an Axio Observer Zeiss microscope. DAPI cells were counted using ImageJ (https://imagej.net/ij/) analyze particle detection and then manually confirmed. Data were collected from three independent experiments.

### 4.14. Animal Behaviour

Foot-fault was used to assess gross motor movement. Mice were placed on an elevated metal grid (1 cm × 1 cm) for 3 min and allowed to freely explore. Video recordings were used to manually score paw slips. Foot-fault slippage ratio was calculated as (number of contralateral paw slips − number of ipsilateral paw slips)/total number of steps × 100. Mice that did not have a change in foot-fault score at PSD3 that was higher than one standard deviation of the mean baseline were deemed not to have a stroke and were excluded from PSD8 analysis. A total of 14 mice per group were tested at PSD3 and PSD8, and 5 mice per group were tested at PSD21.

Gait analysis was performed using CatWalk v10.6 (Noldus, Leesburg, VA, USA). Mice were placed at the entrance of a narrow glass walkway, with a dark goal box containing their home cage on the opposite end of the walkway. As mice walked across the glass walkway to the goal box, paw prints as beam breaks were recorded on a video camera placed underneath the walkway. CatWalkXT software v10.6 (Noldus, Leesburg, VA, USA) was used to analyze print data and obtain quantitative parameters for gait analysis [67]. Seven to ten mice per group were tested on the catwalk. Coupling determines paw placement synchrony between two paws (the length of time between anchor and target steps).

### 4.15. Statistical Analysis

Statistical analysis was conducted using GraphPad Prism6 and v6 SPSS (IBM, Armonk, NY, USA). All immunostained cell counts and neurosphere counts were analyzed with a Student’s *t*-test, unless otherwise stated. NeuN counts were analyzed using a two-way ANOVA, Bonferroni post hoc. Cell viability DAPI counts was analyzed using a two-way ANOVA, Tukey’s post hoc. Foot-fault was analyzed at each test day using a one-way ANOVA, Dunnett post hoc. Catwalk was analyzed using a two-way ANOVA, Bonferroni post hoc. Outliers were removed using the Grubb’s test. All data were represented as mean ± SEM. Significant differences are considered *p* < 0.05 and *p*-values are reported as multiplicity adjusted.

## Data Availability

Data are contained within the article and Supplementary Materials. Additional data presented in this study are available on request from the corresponding author.

## Supplementary Materials

The supporting information can be downloaded at: https://www.mdpi.com/article/10.3390/ijms25031786/s1.
