# Supplementary material for: Inhibition of Apoptosis in a Model of Ischemic Stroke Leads to Enhanced Cell Survival, Endogenous Neural Precursor Cell Activation and Improved Functional Outcomes
Source: Int J Mol Sci. 2024 Feb 1;25(3):1786. doi: 10.3390/ijms25031786 (PMC10855341; doi:10.3390/ijms25031786)
Supplement: Supplementary file 1 [file ijms-25-01786-s001.zip › ijms-2798205-supplementary.pdf]

# Supplementary Table S1

**Table S1.** NWL283 is a selective covalent inhibitor of caspase-3/7. NWL283 has a half maximal inhibitory concentration ($IC_{50}$) of 8 nM against caspase-3 and 21 nM against caspase-7, with minimal inhibition against other caspases.

| **Caspase** | **1** | **2** | **3** | **4** | **5** | **6** | **7** | **8** | **9** | **10** |
|---|---|---|---|---|---|---|---|---|---|---|
| **Enzymatic IC50 (nM)** | 290 | > 10000 | **8** | 27000 | > 10000 | 535 | **21** | 2800 | 41000 | > 10000 |

Supplementary Figure S1

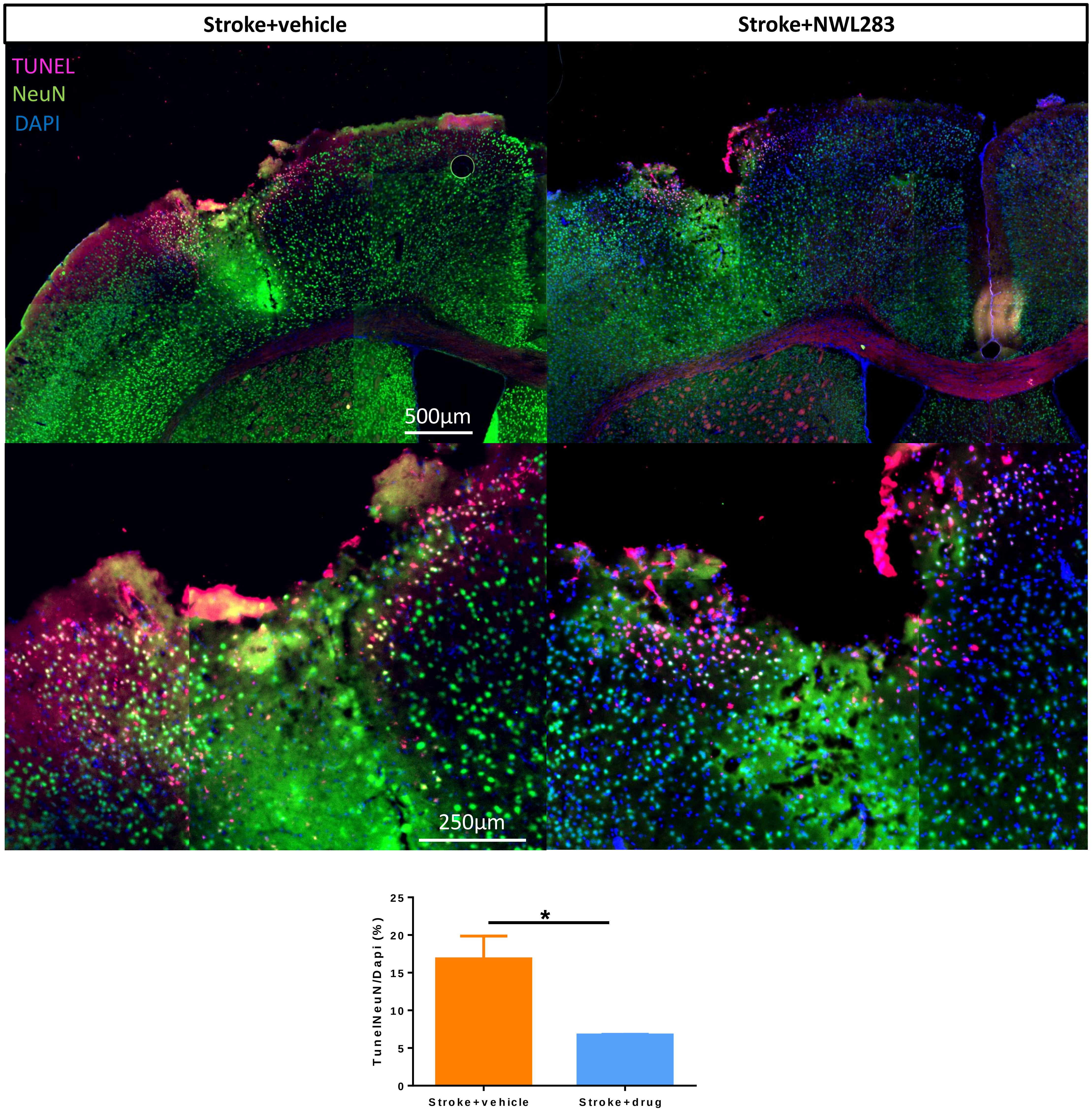


**Figure S1.** NWL283 administration leads to reduced TUNEL+NeuN+ cells. $p$=0.036, Student's T-test. *$p$<0.05, Student's T-test. $n$=3 per group; mean+SEM.

# Supplementary Figure S2

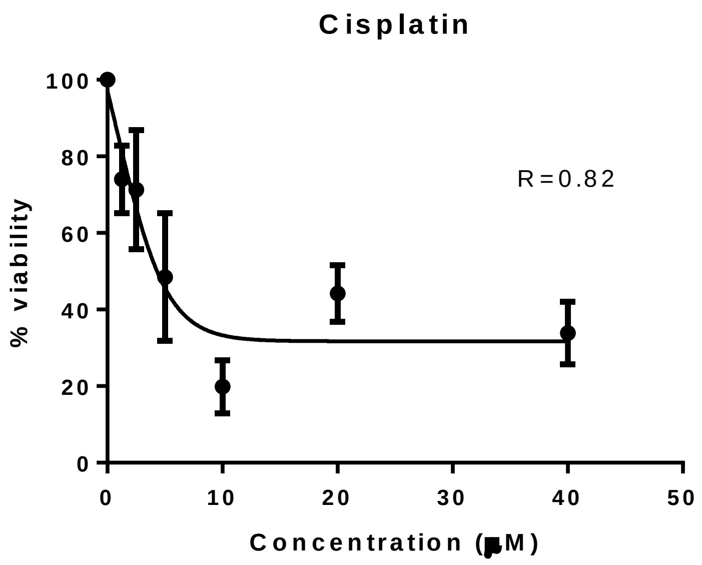


**Figure S2.** NPCs show a cisplatin dose dependent cell death, reaching a plateau at 20µM cisplatin concentration. *n*=3; mean±SEM.

# Supplementary Figure S3

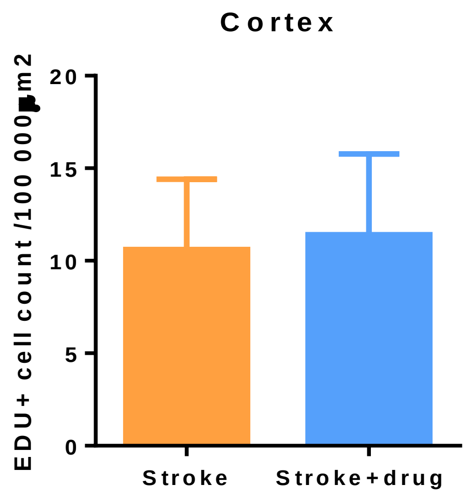


**Figure S3.** The number of EDU+ cells was not different within the cortex of stroke+vehicle and stroke+NWL283 mice. $p$=0.89, Student's T-test. $n$=3-4; mean±SEM.

Supplementary Figure S4

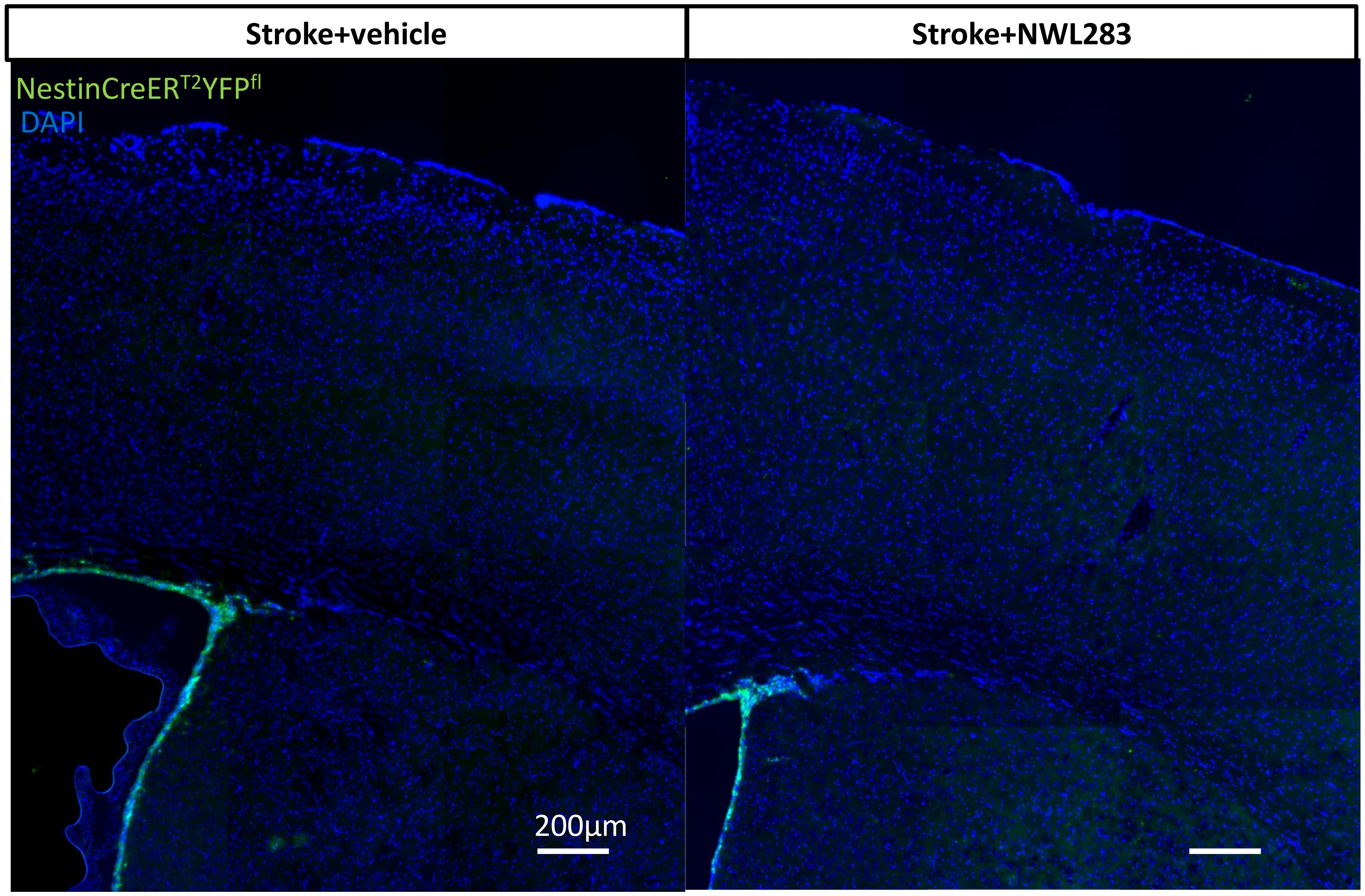


**Figure S4.** NPCs were pre-labelled prior to stroke, using a NestinCreER$^{T2}$YFP$^{fl}$ reporter mouse line. There are no NestinYFP+ cells present in the contralateral hemisphere of stroke+vehicle and stroke+NWL283 mice.
